# Decreased PM_10_ Exposure Attenuates Age-Related Lung Function Decline: Genetic Variants in *p53*, *p21*, and *CCND1* Modify This Effect

**DOI:** 10.1289/ehp.0800430

**Published:** 2009-05-26

**Authors:** Medea Imboden, Joel Schwartz, Christian Schindler, Ivan Curjuric, Wolfgang Berger, Sally L.J. Liu, Erich W. Russi, Ursula Ackermann-Liebrich, Thierry Rochat, Nicole M. Probst-Hensch

**Affiliations:** 1 Department of Chronic Disease Epidemiology, Institute of Social and Preventive Medicine and; 2 Institute of Medical Genetics, University of Zurich, Zurich, Switzerland; 3 Department of Environmental Health, Harvard School of Public Health, Boston, Massachussetts, USA; 4 Institute of Social and Preventive Medicine, University of Basel, Basel, Switzerland; 5 Department of Pneumology, University Hospital Zurich, Zurich, Switzerland; 6 Division of Pulmonary Medicine, University Hospitals Geneva, Geneva, Switzerland

**Keywords:** air pollution, cell cycle, cohort study, genes, respiratory function tests

## Abstract

**Background:**

Decreasing exposure to airborne particulates was previously associated with reduced age-related decline in lung function. However, whether the benefit from improved air quality depends on genetic background is not known. Recent evidence points to the involvement of the genes *p53* and *p21* and of the cell cycle control gene cyclin D1 (*CCND1*) in the response of bronchial cells to air pollution.

**Objective:**

We determined in 4,326 participants of the Swiss Cohort Study on Air Pollution and Lung and Heart Diseases in Adults (SAPALDIA) whether four single-nucleotide polymorphisms in three genes [*CCND1* (rs9344 [P242P], rs667515), *p53* (rs1042522 [R72P]), and *p21* (rs1801270 [S31R])] modified the previously observed attenuation of the decline in the forced expiratory flow between 25% and 75% of the forced vital capacity (FEF_25–75_) associated with improved air quality.

**Methods:**

Subjects of the prospective population-based SAPALDIA cohort were assessed in 1991 and 2002 by spirometry, questionnaires, and biological sample collection for genotyping. We assigned spatially resolved concentrations of particulate matter with aerodynamic diameter ≤ 10 μm (PM_10_) to each participant’s residential history 12 months before the baseline and follow-up assessments.

**Results:**

The effect of diminishing PM_10_ exposure on FEF_25–75_ decline appeared to be modified by *p53* R72P, *CCND1* P242P, and *CCND1* rs667515. For example, a 10-μg/m^3^ decline in aver-age PM_10_ exposure over an 11-year period attenuated the average annual decline in FEF_25–75_ by 21.33 mL/year (95% confidence interval, 10.57–32.08) among participants homozygous for the *CCND1* (P242P) GG genotype, by 13.72 mL/year (5.38–22.06) among GA genotypes, and by 6.00 mL/year (−4.54 to 16.54) among AA genotypes.

**Conclusions:**

Our results suggest that cell cycle control genes may modify the degree to which improved air quality may benefit respiratory function in adults.

A large body of evidence underscores the adverse effect of long-term exposure to ambient particulate matter (PM) air pollution on respiratory health (Brunekreef and Forsberg 2005; Gotschi et al. 2008). Among adults in Switzerland, we have previously demonstrated cross-sectionally that residents of more polluted areas have lower lung function (Ackermann-Liebrich et al. 1997). More recently, we presented evidence from the same population-based cohort [Swiss Cohort Study on Air Pollution and Lung and Heart Diseases in Adults (SAPALDIA)] that decreasing exposure to airborne PM attenuated the average age-related decline in lung function. The associations were strongest for respiratory function tests reflecting small-airway function, namely, FEF_25–75_ [forced expiratory flow between 25% and 75% of forced vital capacity (FVC)] (Downs et al. 2007). Similar results from studies following interventions such as building bypasses for congested traffic routes (Burr et al. 2004; Hedley et al. 2002) or banning environmental tobacco smoke (ETS) exposure (Goodman et al. 2007; Menzies et al. 2006) showed that the improvements in air quality were accompanied by a decrease in cardiopulmonary mortality and an improvement in respiratory symptoms and lung function. However, it is still unknown whether all subjects benefit equally from a reduction in air pollution.

Variation in genes mediating the pathobiological effect of air pollution in the lung may codetermine the degree to which a person benefits from better air quality. Experimental evidence indicates that PM alters expression of tumor protein gene *p53*, cyclin-dependent kinase inhibitor 1A gene (*p21*), and the cyclin D1 gene (*CCND1*) and subsequently affects cell proliferation and apoptosis of lung fibroblasts, lymphocytes, and alveolar epithelial cells (Bayram et al. 2006; Dagher et al. 2006; Nyunoya et al. 2006; Rosas Perez et al. 2007; Soberanes et al. 2006). PM is furthermore well known to induce oxidative stress in the airways (Li et al. 2008). In fact, the expression of all three gene candidates, *p53*, *p21*, and *CCND1*, in bronchial epithelial cells and lung fibroblasts seems to be regulated in part by redox-dependent mechanisms (Jiao et al. 2008; Ranjan et al. 2006; Yao et al. 2008).

The tumor suppressor p53, a nuclear transcription factor, binds to response elements in the promoter region of many genes and plays a pivotal role in apoptosis. It induces up-regulation of the expression of many pro-apoptotic genes and down-regulation of anti-apoptotic genes (Oren et al. 2002). CCND1 (cyclin D1) is known to promote cell proliferation through cell cycle G1–S phase transition. The protein p21 (also known as Waf1 or Cip1) is a direct functional counterpart of CCND1 and an important downstream effector of p53 action that negatively regulates cell proliferation. *CCND1*, *p21*, and *p53* all harbor polymorphisms of hypothesized functional relevance that have been extensively studied in the context of cancer (Choi et al. 2008; Lu et al. 2008; Zhou et al. 2007). In this study, we examined whether these polymorphisms modified the degree to which the age-related FEF_25–75_ decline was attenuated by reduced exposure to PM with aerodynamic diameter ≤ 10 μm (PM_10_).

## Materials and Methods

### SAPALDIA cohort study population

The study design and methodology of SAPALDIA have been described in detail elsewhere (Ackermann-Liebrich et al. 2005; Martin et al. 1997). In 1991, health examinations focusing on respiratory health status were conducted in 9,651 adults (18–60 years of age) randomly selected from population registries of eight environmentally diverse areas of Switzerland. Ethical approval was obtained from the Swiss Academy of Medical Sciences and the Regional Ethics Committees. Written informed consent was obtained from all participants before health examination and biological sample collection at both surveys. Nonparticipation at follow-up and missing information on covariates led to the exclusion of participants for the present study [see Supplemental Material, Figure 1 and Table 2 available online (doi:10.1289/ehp.0800430. S1 via http://dx.doi.org)]. In summary, of the 9,651 subjects who initially participated at baseline (SAPALDIA1), 1,604 had died, left Switzerland, or refused to participate at the follow-up examination (SAPALDIA2). Of 8,047 cohort participants remaining, 5,732 completed the interview questionnaire and spirometry at both the baseline and follow-up surveys. Participants were excluded from the analysis (*n*= 1,406) if they had lived for < 1 year at their last residential address at follow-up, could not be assigned home outdoor PM_10_ concentrations, or did not provide blood samples for DNA extraction or if the genetic analysis of their sample was unsuccessful. Thus, the present study sample included 4,326 participants with available blood samples and genotype data and complete data from both surveys on spirometry, smoking history, PM_10_ exposure, and residential history during follow-up.

### Lung function assessment

Identical spirometer devices (model 2200, SensorMedics Corp., Yorba Linda, CA, USA) and protocols were used at baseline and follow-up examinations, and their comparability was assessed before the follow-up study (Künzli et al. 1995, 2005). Details of these measurements have been described elsewhere (Ackermann-Liebrich et al. 2005; Downs et al. 2005). Briefly, three to eight forced expiratory lung function maneuvers were performed by each participant to obtain a minimum of two measures of FEF, FVC, forced expiratory volume in the first second (FEV_1_), and FEF_25–75_ that were considered acceptable according to American Thoracic Society (1995) criteria. Expiratory flow measures were taken from the same flow-volume curve. For each participant, the rate of change in lung function was defined as the difference in each parameter between the two examinations (measurement at follow-up minus measurement at baseline), divided by the participant-specific follow-up time (in nontruncated years).

### Selection of genetic variants

We selected the same common, well-studied, and potentially functional candidate single-nucleotide polymorphism (SNP) in *CCND1* [proline-to-proline substitution at amino acid 242 (P242P), rs9344] that we evaluated in our previous research on the interaction of *CCND1* with oxidative stress in breast and colon cancer (Ceschi et al. 2005; Probst-Hensch et al. 2006). We selected one additional common SNP in the *CCND1* gene (rs667515) based on a pilot study involving haplotype-tagging polymorphisms in *CCND1* and their association with accelerated lung function decline in a subsample of the SAPALDIA cohort (unpublished observations). In addition, we examined SNPs in two other important cell cycle control genes that have been repeatedly assessed in cancer association studies and are reported to have a functional effect: *p53* [arginine-to-proline substitution at amino acid 72 (R72P), rs1042522] (Bergamaschi et al. 2006) and *p21* [serine-to-arginine substitution at amino acid 31 (S31R), rs1801270](Lukas et al. 1997).

### Genotpying

DNA was extracted from EDTA-buffered whole blood as previously described (Ackermann-Liebrich et al. 2005). Genotyping strategy used was fluorescent 5′-nuclease real-time polymerase chain reaction (TaqMan, Applera Europe, Rotkreuz, Switzerland) methodology using ABI Prism 7900 sequence detection system (ABI, Rotkreuz, Switzerland). The SNP-specific primers and locked nucleic acid (LNA) dual-labeled fluorogenic probes were designed by Sigma Proligo (Evry, France). SNP-specific probes and primers were as follows: rs1042522, 5′-CTGCTCCCCuC/G3CGTGGC-3′, forward 5′-ACTGAAGACCCAGGTCCA-3′, reverse 5′-GCCGGTGTAGGAGCT-3′; rs1801270, 5′-GCTGAGuC/A3CGCGAC-3′, forward 5′-TGCCGCCGCCTCTT-3′, reverse 5′-GATGCAGCCCGCCATTAG-3′; rs667515, 5′-AGCTCCCTTGCuG/C3 CCC-3′, forward 5′-TGGCTTCAT_ C A G A T G A C A A C -3′, reverse 5′-AACCTGGGCTTCTCCAA-3′; rs9344, 5′-TGTGACCCuA/G3GTAAGTGA-3′, forward 5′-ACGCTTCCTCTCCAGAG_3′, reverse 5′-CAAGGCTGCCTGG-3′. A 10% random sample of all DNA samples was regenotyped, and all geno types were confirmed. The genotype call rate was > 99%.

### Individual residential PM_10_ exposure assessment

Air pollution exposure assessment has been described in detail elsewhere (Downs et al. 2007; Liu et al. 2007). We estimated PM_10_ exposure for each participant according to residential history using a hybrid exposure model incorporating Gaussian dispersion (from PolluMap model, version 2.0; Liu et al. 2007) and geoinformation based on data on seasonal, meteorologic, and geographic annual emission characteristics from various source categories (e.g., traffic, industrial, regional, and agricultural activities) (Liu et al. 2007). Hourly concentrations of PM_10_ were simulated with a spatial resolution of 200 × 200 m grid cells for 1990 and 2002. Annual average PM_10_ exposure concentrations during follow-up were estimated for each residential address with the help of an algorithm that allowed interpolation of modeled values on the basis of historical trends in central-site measurements between 1990 and 2002. Evaluation of dispersion model predictions using a total of 57 PM_10_ Swiss central-site monitors has been described elsewhere (Liu et al. 2007). Based on the validated dispersion model, each subject was assigned an annual PM_10_ concentration every year between 1990 and 2002. For that purpose, modeled PM_10_ concentrations were averaged over the year to obtain annual averages for each grid cell. Each participant’s residential history was coded using the geographic information system data. Assignment of individual PM_10_ exposure was performed by matching address codes with annual concentrations derived from the grid cells generated by the dispersion model.

The exposure variable used for the present study was the difference in the annual average PM_10_ exposure between 2002 and 1991. It was calculated for each participant as follows: average home outdoor PM_10_ concentration in the 12 months before the baseline examination was subtracted from the corresponding average concentration in the 12 months before the follow-up examination, and this difference was divided by the mean time of follow-up (in nontruncated years). In our previous study (Downs et al. 2007), we used two exposure indexes: *a*) the difference in the annual average exposure between 2002 and 1991 (the index used in the present study ) and *b*) the “interval exposure,” defined as the sum of the annual exposures for each subject for each year of follow-up between their examinations. Because of the high correlation (*R*^2^ > 0.9) between these two exposure variables, we were unable to single out the superior measure in our previous report. Both indices showed similar associations with change in lung function (Downs et al. 2007).

### Collection of data on covariates

Information on relevant covariates known or likely to determine lung function decline, such as age, height, smoking history, ETS exposure, occupational exposure, and education level, was gathered in a computer-assisted, individually administered interview based on the European Community Respiratory Health Survey questionnaire (Burney et al. 1994). Current and past smoking habits, exposure to ETS, and occupational exposure to dust and fumes were assessed with the same questions at both surveys. Participants who reported smoking < 20 packs of cigarettes and using < 360 g of tobacco in their lifetime at both time points were defined as never-smokers. Cumulative cigarette exposure of participants was assessed by pack-years smoked before the first examination and pack-years smoked during follow-up. Participants were asked not to smoke in the hour before the examination, and smoking was validated by measuring the carbon monoxide concentration in exhaled breath using an EC 50 Micro-Smokerlizer (Bedfont Scientific, Rochester, UK). Atopic status was assessed at baseline using skin prick tests (Phazet; Pharmacia, Uppsala, Sweden) for eight common inhalant allergens: dog epithelium; cat fur; pollen of timothy grass, *Parietaria*, and birch; the house dust mite *Dermatophagoides pteronyssinus*; and the molds *Alternaria tenuis* and *Cladosporium herbarum* (Martin et al. 1997; Wuthrich et al. 1996). Atopy was defined by an adjusted mean wheal diameter ≥ 3 mm to at least one allergen.

### Statistical analysis

We inferred *CCND1* haplotypes from unphased genotype data using PHASE 2.1 algorithm software [see Supplemental Material, Table 1 (doi:10.1289/ehp.0800430.S1)] (Stephens et al. 2001). Hardy-Weinberg equilibrium was tested using STATA gtab command for global κ-statistic testing (STATA version 10; StataCorp, College Station, TX, USA). All four SNPs were in Hardy-Weinberg equilibrium. We obtained Lewtonin’s linkage disequilibrium (LD) metric D′using STATA command pwld for pairwise LD. Descriptive analyses of the lung function parameters, PM_10_, smoking, socioeconomic variables, and other relevant covariates have been described previously and in detail in different SAPALDIA cohort study reports (Ackermann-Liebrich et al. 2005; Downs et al. 2007; Imboden et al. 2007). We compared baseline characteristics of the cohort participants included in this study (*n* = 4,326) with subjects participating only at baseline as well as with cohort participants excluded from this analysis because information on genotype or covariate data was missing.

Results are presented as the estimated effect of a 10-μg/m^3^ decrease in the annual average PM_10_ exposure over the follow-up period (ΔPM_10_) on the average attenuation of the annual decline in FEF_25–75_.

The association between ΔPM_10_ and the average annual rate of lung function decline had been previously assessed using mixed linear model analysis, and selection of relevant covariates was based on this previous investigation (Downs et al. 2007): age at baseline (SAPALDIA1), age^2^, sex, height, parental smoking during childhood reported at baseline, sine and cosine function of day of examination to control for seasonal effects, level of education at baseline, change in level of education, Swiss nationality, self-reported occupational exposure to dust and occupational exposure to fumes at SAPALDIA1 and SAPALDIA2 (yes/no), smoking status at follow-up (never, former, or current), pack-years up to SAPALDIA1, pack-years between SAPALDIA1 and -2, cigarettes per day at SAPALDIA1 and -2, atopy, body mass index (BMI) at SAPALDIA1, change in BMI, interaction between the two BMI variables, and baseline PM_10_ exposure. Random effects were included to adjust for clustering of residuals within area and were assumed to be independent between the areas and to have an exchangeable correlation structure. To estimate main effects of gene variants of *CCND1*, *p21*, and *p53* and rate of lung function decline, we used the same mixed model with random area effects additionally adjusted for ΔPM_10_. To estimate modification of the ΔPM_10_ effect on average lung function decline by genotype, we introduced interaction terms between ΔPM_10_ and genotypes into the above-described covariate-adjusted mixed linear models independently for each SNP. Also, for each SNP we evaluated three different genetic models (codominant, dominant, and recessive) because previous cancer association studies were not conclusive about the underlying genetic model of the SNP effects. The codominant model assumes that the gene effect depends on the number of alleles in a dose-dependent manner, the dominant model assumes that the gene effect depends on the presence of at least one of two high-risk alleles, and the recessive model assumes that the gene effect depends on the presence of both high-risk alleles. The three genetic models required different coding of genotypes. For each SNP we present the genetic model with the smallest interaction term *p*-value. We obtained effect estimates for the association between ΔPM_10_ and average annual lung function decline in genotype subgroups by creating genotype- specific PM_10_ exposures variables that we introduced into separate covariate-adjusted mixed linear models. Analyses were conducted using SAS release 9.1 (SAS Institute Inc., Cary, NC, USA) and STATA version 10. *p*-Values < 0.05 were interpreted as statistically significant for main and interaction effects. *p*-Values presented as main *p*-values in the tables are not corrected for multiple testing. Nevertheless, Bonferroni-corrected significance level (α = 0.05 divided by the number of tests) is indicated in tables and figures as appropriate.

## Results

### Population characterization

Table 1 describes the general characteristics of the study population (*n* = 4,326), including relevant predictors of lung function. This is a population consisting mostly of Caucasians. At follow-up participants were more likely to be women and less likely to be smokers than were non-participants. Participants tended to gain weight, give up smoking, and reduce exposure to dust or fumes at work or to ETS [for details, see Supplemental Material, Table 2 (doi:10.1289/ehp.0800430.S1)].

In 2002, 87% of the participants were living in the same area as in 1991, and 54% had the same address. In general, individual home outdoor concentrations of PM_10_ declined during the follow-up period (Table 1), as previously described in detail (Downs et al. 2007; Liu et al. 2007). The median decline between examinations for subjects included in this analysis was 5.8 μg/m^3^ (interquartile range, 4.2–7.3 μg/m^3^). Mean decline was greatest for participants living in urban areas and lowest in alpine areas.

Mean lung function for the cohort was within the range of predicted values for the general population and declined during the follow-up period (Table 1). The mean ± SD annual change in mid FEF_25–75_ during the 11-year follow-up period was −74.1 ± 70.3 mL/year in men and −68.9 ± 58.9 mL/year in women included in this analysis.

The main effects of the investigated genetic variants on change in lung function are described in more detail in Supplemental Material, Table 3 (doi:10.1289/ehp.0800430. S1). Briefly, we observed no statistically significant associations of the polymorphisms either with change in FEF_25–75_ or with FEV_1_ or FVC.

### Modification of the ΔPM_10_ effect on aver-age decline in lung function by genotype

**Figure 1:**
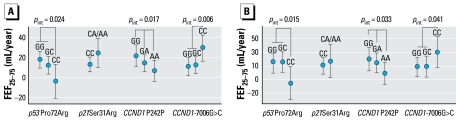
Attenuation of average annual FEF_25–75_ decline associated with a 10-μg/m^3^ decrease in average home outdoor PM_10_ exposure between 1991 and 2002, by genotype status, in all study participants (*A*) and in never-smokers only (*B*). A positive value for FEF_25–75_ on the *y*-axes represents the average attenuation in lung function decline associated with an average 10-μg/m^3^ PM_10_ decrease during follow-up period. Bonferroni significance level for four comparisons *p* = 0.013.

As previously reported (Downs et al. 2007), a 10-μg/m^3^ decline in average annual home outdoor PM_10_ concentration over an 11-year period (ΔPM_10_) reduced the annual rate of decline in FEF_25–75_ on average by 11.2 mL/year or 16%. Here we report statistically signifi cant modifications of this association of ΔPM_10_ with FEF_25–75_ decline by three of the four genetic variants investigated: *CCND1* P242P [*p*-value for interaction (*p*_int_) = 0.017], *CCND1* rs667515 (*p*_int_ = 0.006), and *p53* R72P (*p*_int_ = 0.016). Figure 1A and Table 2 present results for the association of ΔPM_10_ with the aver-age annual change in FEF_25–75_ within genotype and diplotype strata for the entire study population; for data for never-smokers, see Figure 1B and Supplemental Material, Table 4 (doi:10.1289/ehp.0800430.S1). Equivalent to the previously reported main effect of ΔPM_10_ (Downs et al. 2007), the genotype-specific results we observed in the entire study popu lation were comparable to those in never-smokers. The A allele for *CCND1* P242P reduced the attenuating effect estimate of ΔPM_10_ on FEF_25–75_ decline in a codominant manner. A 10 μg/m^3^ decline in PM_10_ over an 11-year period was associated with an average attenuation of annual decline in FEF_25–75_ of 6.0 mL/year [95% confidence interval (CI), −4.54 to 16.54] in participants with an AA genotype, 13.7 mL/year (5.38–22.06) in heterozygous AG participants, and 21.3 mL/year (10.57–32.08) in participants with a GG genotype.

For the *CCND1* rs667515 SNP, the beneficial ΔPM_10_ effect was most pronounced in participants homozygous for the minor allele (CC genotype) and attenuated annual FEF_25–75_ decline in this subgroup by 28.83 mL/year (95% CI, 15.60–42.07) compared with 10.39 (3.24–20.47) and 11.83 (1.32–19.47) among GG and GC genotypes, respectively. This effect modification thus followed a recessive genetic model.

For the *p53* R72P SNP, the observed effect modification followed a codominant model. ΔPM_10_ was associated with an aver-age attenuation of annual FEF_25–75_ decline by an average of 17.4 mL/year (95% CI, 8.95–25.78) in GG genotypes (Pro/Pro) and by an average of 11.6 mL/year (2.65–20.61) in CG genotypes (Arg/Pro) but no attenuation in CC genotypes (Arg/Arg).

*CCND1* haplotypes 2 and 3, but not 1 and 4, were also statistically significant modifiers of the ΔPM_10_ association with FEF_25–75_ decline. In participants exhibiting the *CCND1* haplotype 3 (CG/CG) on both alleles (representing the combination of the single SNP alleles associated with the greatest lung function attenuation), the effect estimate for attenuation of FEF_25–75_ decline associated with ΔPM_10_ was 30.9 mL/year (95% CI, 17.21–44.58) compared with 11.92 (3.40–20.44) and 10.28 (1.22–19.35) among subjects with the CG haplotype in one or none of their alleles (Table 2).

Genotype-specific ΔPM_10_ effect estimates for the attenuation of the average annual decline in FEV_1_ and FVC are presented in Supplemental Material, Table 5 (doi:10.1289/ehp.0800430.S1). For FEV_1_ decline, effect modification by genotypes was comparable to that seen for FEF_25–75_ decline. Again, effect modification was strongest for the *CCND1* rs667515 SNP and for *CCND1* haplo-type3 [see Supplemental Material, Table 5 (doi:10.1289/ehp.0800430.S1)] We observed no modification of the ΔPM_10_ effect on FVC decline for any of the SNPs or haplotypes.

## Discussion

Understanding the pathways by which PM_10_ damages lung structures and identifying susceptible subjects are important steps in managing the public health challenges of anthropogenic ambient air pollution. In this study, we present novel evidence that genetic polymorphisms in the cell fate controlling genes, *p53*, *p21*, and *CCND1* may modify the degree to which adults benefit from improved air quality. If confirmed by independent studies, the results are of public health relevance. The observed difference between genotype subgroups with regard to ΔPM_10_ effects on average lung function decline ranged between 11 mL/year and 21 mL/year for FEF_25–75_. This difference compares with the observed excess mean annual decline in FEF_25–75_ of 18 mL and 14 mL per pack per day smoked during follow-up by, respectively, male and female SAPALDIA participants who smoked at baseline (modeled according to Downs et al. 2005). This difference in lung function decline may seem small in absolute terms and from an individual perspective, but even slight shifts in the population distribution of lung function can substantially increase the prevalence of subjects exhibiting respiratory function below clinical thresholds. In addition, lung function is known to be a strong and independent predictor of overall mortality (Künzli et al. 2000a, 2000b).

The mechanisms by which air pollutants induce harmful changes in bronchial tissue and ultimately lead to respiratory symptoms and clinically relevant respiratory dysfunction have been investigated. It seems well established that PM directly increases oxidative stress (Donaldson et al. 2003), activates the expression of proinflammatory cytokines (e.g., tumor necrosis factor α, nuclear factor-κβ, and interleukin-8) (Rahman et al. 2002) and induces the homing of inflammatory cells such as neutrophils, alveolar macrophages, and dendritic cells, thus resulting in inflammation of the lung (Behndig et al. 2006; Bosson et al. 2008; Li et al. 1996,^1997^; Nightingale et al. 2000). The toxic effects of PM are further enhanced by bronchial tissue injuries and increased epithelial permeability (Morrison et al. 1999).

Our results are in line with more recent *in vitro* experiments with different types of bronchial cell lines demonstrating that PM induces cell proliferation arrest and apoptosis in exposed cells (Bayram et al. 2006; Dagher et al. 2006; Rosas Perez et al. 2007; Soberanes et al. 2006; Upadhyay et al. 2003; Zhang et al. 2007). Air pollutants or cigarette smoke, both exhibiting oxidative properties, specifically alter the expression levels of tumor suppressor genes such as *p53* and *p21* and of the cell cycle control gene *CCND1* in human and rodent *in vitro* bronchial cell culture models. Although *p53* and *p21* expression has been consistently shown to be increased upon PM exposure (Bayram et al. 2006; Dagher et al. 2006; Jiao et al. 2008; Nyunoya et al. 2006; Palozza et al. 2006; Rosas Perez et al. 2007; Soberanes et al. 2006; Yao et al. 2008; Zhang et al. 2007), *CCND1* expression has been reported to be either inhibited (via *p21* activation) (Palozza et al. 2006; Zhang et al. 2007) or increased [*p53*-driven N-terminal C-Jun kinase (*cJUN*) activation] (Bayram et al. 2006; Dagher et al. 2007; Jiao et al. 2008). Such variations in the expression of cell cycle genes mediate the adaptive cellular response to changes in environmental exposure. They might well be the starting point of PM_10_-induced pathologic changes in morphology and in the type and number of lung fibroblasts and bronchial epithelial cells. Activation of cell proliferation processes may help to maintain intact airways even in the presence of environmental toxins, as suggested by genetic ablation of *p21* in mice, which conferred protection against cigarette-smoke–induced lung inflammation and injury (Yao et al. 2008). Aspects of bronchial tissue remodeling represent key features of pathologic changes in the etiology of most airway disorders. However, how proliferation and apoptosis relate to airway remodeling in general remains poorly understood and is likely to be disease specific.

Three of the four polymorphisms investigated here have been studied before, mainly in cancer studies, and allelic functional differences have been proposed. The functional consequence of *p53* R72P polymorphism was studied in detail. The Arg72-allele (G-allele) of *p53* is more active at inducing apoptosis than is the *p53* Pro72-allele. In fact, Pro72 is part of a PXXP motif known to be critical in binding of a *p53*-specific inhibitory protein, iASPP (inhibitory member of the apoptosis- stimulating protein phosphatase family) (Bergamaschi et al. 2006). Along the line of the proposed functional consequence of this genetic variant, meta-analysis results have recently shown that the homozygous Pro/Pro genotype showed a proproliferative effect and was associated with increased gastric cancer risk (Zhou et al. 2007). In our association study, we observed the most pronounced benefits of ΔPM_10_ with regard to FEF_25–75_ decline in subjects with one or two Arg72-alleles, which are expected to induce apoptosis more potently than the Pro72-allele. In contrast, homozygous carriers of the Pro72-allele did not appear to benefit from the improvement in air quality.

The functional studies of the common *CCND1* polymorphism P242P showed a modification of the alternative splicing events in exon 4 because the G-to-A substitution alters the consensus sequence of the splicing donor site (Betticher et al. 1995; Howe and Lynas 2001). As recently reported by three meta-analyses, the homozygous AA genotype was associated with increased risk of various types of cancer (Lu et al. 2008; Pabalan et al. 2008; Tan et al. 2008). The A-allele of the *CCND1* 870G>A (P242P) polymorphism, a proposed genetic risk factor for lung cancer, was previously associated with impaired cell cycle regulation and accumulation of DNA damage in the airway (Buch et al. 2005; Munnia et al. 2006). In our association analysis, we observed that homo- or heterozygous G-allele carriers benefited more from the decrease in PM_10_ exposure than did homozygous A-allele carriers. The association of the second *CCND1* (−7006G>C) SNP with cancer or any other outcome has not been investigated previously. Its functional consequence, if any, might be regulatory because it is situated upstream of the *CCND1* gene. But it is also possible that this SNP is in high linkage with a yet unknown functional *CCND1* variant, just as it is also in high LD with the *CCND1* P242P variant.

The polymorphism S31R in the *p21* gene was shown to be located in the DNA-binding zinc finger motif and thus has been thought to alter the function of the *p21* protein (Lukas et al. 1997). However, results of cancer studies for this SNP have been inconsistent with regard to the size and direction of relative risk estimates (Choi et al. 2008), and we observed no interaction between this SNP and ΔPM_10_ on lung function decline.

Thus, the cancer-promoting *p53* and *CCND1* alleles seem to reduce the benefit of improved air quality on respiratory function. Only limited epidemiological data exist on the association of *p53*, *p21*, or *CCND1* SNPs with respiratory diseases such as asthma, emphysema, and chronic obstructive pulmonary disease (COPD). The polymorphisms we evaluated in *p53* and *p21* have previously been associated with smoking-related COPD. Compared with healthy smokers, the cancer risk allele of *p53* (Pro72-allele), and the *p21* Arg31-allele were overrepresented in COPD patients (Lee et al. 2006). The extrapolation of observed associations with cancer to expected associations with lung function is not straightforward. The present findings and our interpretation of them should be considered as exploratory in nature and need confirmation in independent studies. The relative impacts of cell proliferation and apoptosis on different cell types in the lung and on respiratory function must be further investigated by experimental studies.

The strengths of the present study are its prospective design, its rather large sample size, and detailed characterization of the study participants, as well as the availability of individual air pollution exposure history since 1990. We were thus able to adjust lung function decline for most of the potential confounders. Nonetheless, information on some relevant confounders such as dietary antioxidant intake and physical activity was collected only at follow-up. Lacking data on the degree of genetic admixture of the Swiss general population is an additional drawback. However, we expect little bias due to genetic admixture, because the prevalence of several polymorphisms studied in the cohort did not vary across the three major Swiss language groups. We assessed a very limited number of carefully selected candidate polymorphisms; for some of the SNPs and haplotypes, and especially for the *p21* SNP and *CCND1* haplotype 4, statistical power to detect effect estimation was very limited. A more comprehensive assessment of gene variants in the apoptosis pathway and genes regulating apoptosis in lung tissue needs to be addressed in future larger studies. Nevertheless, even for the small number of genetic variants investigated, multiple testing represents a limitation in the interpretation of the present results. Consistency of the associations across genes involved in cell cycle control, as well as across various lung function outcomes, diminishes the likelihood of chance findings, as does the strength of the observed *p*_int_ values.

Finally, participation at follow-up and in this present study was not complete, so participation bias cannot be excluded. However, because we observed comparable results in all subjects and in never-smokers and in the absence of different effects between men and women, the lower participation rate in men and smokers is not likely to have caused bias.

In conclusion, our results suggest that even at low to moderate levels of air pollution, such as in Switzerland, some but not all persons benefit from improved air quality. Given the novelty of the finding and the limitations inherent to this study, the results need independent confirmation. Future studies must address additional issues. First, gene variants relevant to cell cycle control must be studied more comprehensively. Second, it is of interest to know whether this novel candidate pathway might determine susceptibility to both improvements and declines in air quality.

## Figures and Tables

**Table 1:** Characteristics of the study population: SAPALDIA cohort.

Characteristic	Participants (*n* = 4,326)
Female (%)	53.0
Swiss nationality (%)	87.7
Educational level in 2002 (professional education or higher, %)	27.9
Increase in educational level between surveys (%)	17.9
Age in 1991 [mean ± SD (years)]	41.3 ± 11.2
Height [mean ± SD (cm)]	169.3 ± 8.8
BMI in 1991 [mean ± SD (kg/m^2^)]	23.7 ± 3.6
BMI change [mean ± SD (kg/m^2^)]	2.1 ± 2.2
Smoking	
Never-smokers in 1991 (%)	48.4
Never-smokers in 2002 (%)	49.3
Smoking quitters during follow-up (%)	8.1
Current smokers in 1991 (%)	29.3
Current smokers in 2002 (%)	21.9
No. of pack-years for current smokers in 2002 [median (IQR)]	26.7 (14.0–42.6)
Cigarettes per day for current smokers in 1991 [median (IQR)]	20 (10–25)
Cigarettes per day for current smokers in 2002 [median (IQR)]	15 (7–20)
Passive smoking and occupational exposure (%)	
ETS exposure in never-smokers in 1991	13.1
ETS exposure in never-smokers in 2002	7.7
Father or mother smoked during childhood	56.1
Workplace exposure to dust and fumes in 1991	30.3
Workplace exposure to dust and fumes in 2002	13.2
Atopy in 1991	22.3
Change in average individual home outdoor PM_10_ exposure^a^	
All areas (*n* = 4,326)	−5.8 (−7.3 to −4.2)
Basel area (*n* = 486)	−8.0 (−9.1 to −6.9)
Wald area (*n* = 889)	−4.5 (−4.8 to −3.9)
Davos area (*n* = 318)	−3.0 (−3.1 to −2.8)
Lugano area (*n* = 568)	−12.1 (−13.5 to −10.8)
Montana area (*n* = 431)	−4.0 (−4.2 to −3.7)
Payerne area (*n* = 595)	−5.0 (−5.3 to −4.6)
Aarau area (*n* = 689)	−6.4 (−6.8 to −5.8)
Geneva area (*n* = 350)	−6.2 (−7.3 to −5.7)
Lung function in 1991 [mean ± SD (L)]^b^	
FVC in women	3.82 ± 0.61
FVC in men	5.30 ± 0.82
FEV_1_ in women	3.07 ± 0.55
FEV_1_ in men	4.11 ± 0.72
FEF_25–75_ in women	3.07 ± 1.00
FEF_25–75_ in men	3.79 ± 1.29
Annual change [mean ± SD) (mL/year)]^b^	
FVC in women	−20.79 ± 34.11
FVC in men	−29.17 ± 45.47
FEV_1_ in women	−31.88 ± 25.72
FEV_1_ in men	−39.77 ± 32.91
FEF_25–75_ in women	−68.89 ± 58.88
FEF_25–75_ in men	−74.11 ± 70.24
Genotype distribution	
*p53* P72R, rs1042522	
GG	2,407
CG	1,637
CC	282
*p21* S31R, rs1801270	
CC	3,701
CA or AA^c^	625
*CCND1* P242P, rs9344	
GG	1,211
GA	2,140
AA	975
*CCND1* −7006G>C, rs667515	
GG	1,628
GC	2,058
CC	640
Diplotype distribution^d^	
*CCND1* haplotype 1 (rs667515, rs9344)
−/−	3,125
GG/−	1,088
GG/GG	113
*CCND1* haplotype 2 (rs667515, rs9344)	
−/−	1,251
GA/−	2,150
GA/GA	925
*CCND1* haplotype 3 (rs667515, rs9344)	
−/−	1,678
CG/−	2,048
CG/CG	600
*CCND1* haplotype 4 (rs667515, rs9344)	
−/−	4,239
CA/−	84
CA/CA	3

IQR, interquartile range.

a PM_10_ (μg/m^3^) in the year before SAPALDIA1 minus PM_10_ in the year before SAPALDIA2.

b Women, *n* = 2,293; men, *n* = 2,033.

c Genotype distribution: *p21* CA, *n* = 594; *p21* AA, *n* = 31.

d Diplo-type distribution is labeled as follows: −/−, none of the specific haplotype present; (*rs667515*, *rs9344*)/− (e.g., GG/−), one of the specific haplotypes present; (*rs667515*, *rs9344*)/(*rs667515*, *rs9344*) (e.g., GG/GG), for two of the specific haplotypes present.

**Table 2:** Effect modification by genotypes: association^a^ of change in average home outdoor PM_10_ (per decrease of 10 μg/m^3^ between 1991 and 2002) with average annual decline in FEF_25–75_ (mL/year), by genotype status.

Genotype	No.	Average annual FEF_25–75_ decline^b^	95% CI	*p*-Value	*p*_int_^c^
*p53* R72P; rs1042522					
GG	2,407	17.37	8.95 to 25.78	< 0.001	0.016_(codominant)_
CG	1,637	11.63	2.65 to 20.61	0.011	
CC	282	−4.33	−21.37 to 12.7	0.618	
*p21* S31R; rs1801270					
CC	3,701	12.72	5.35 to 20.1	0.001	0.115_(dominant)_
CA or AA^d^	625	23.88	9.25 to 38.51	0.001	
*CCND1* P242P; rs9344					
GG	1,211	21.33	10.57 to 32.08	< 0.001	0.017_(codominant)_
AG	2,140	13.72	5.38 to 22.06	0.001	
AA	975	6.00	−4.54 to 16.54	0.265	
*CCND1* −7006G>C; rs667515					
GG	1,628	10.39	1.32 to 19.47	0.025	0.006_(recessive)_
CG	2,058	11.83	3.24 to 20.42	0.007	
CC	640	28.83	15.6 to 42.07	< 0.001	
*CCND1* haplotype 1 (rs667515, rs9344)^e^					
−/−	3,125	13.67	5.99 to 21.34	< 0.001	0.156_(recessive)_
GG/−	1,088	11.1	0.26 to 21.94	0.045	
GG/GG	113	37.77	3.24 to 72.3	0.032	
*CCND1* haplotype 2 (rs667515, rs9344)^e^					
−/−	1,251	20.66	10.07 to 31.24	< 0.001	0.022_(codominant)_
GA/−	2,150	13.75	5.34 to 22.15	0.001	
GA/GA	925	6.08	−4.5 to 16.67	0.26	
*CCND1* haplotype 3 (rs667515, rs9344)^e^					
−/−	1,678	10.28	1.22 to 19.35	0.026	0.003_(recessive)_
CG/−	2,048	11.92	3.4 to 20.44	0.006	
CG/CG	600	30.9	17.21 to 44.58	< 0.001	
*CCND1* haplotype 4 (rs667515, rs9344)^e^					
−/−	4,239	13.52	6.22 to 20.82	< 0.001	0.434_(codominant)_
CA/−	84	−1.4	−39.67 to 36.88	0.943	
CA/CA	3	−11.8	−468.22 to 444.62	0.96	

a Covariates were age, age^2^, sex, height, parental smoking, sine and cosine function of day of examination to control for seasonal effects, level of education at SAPALDIA1, change in level of education, Swiss nationality, self-reported occupational exposure to dust and occupational exposure to fumes at SAPALDIA1 and SAPALDIA2 (yes/no), smoking status at SAPALDIA2 (never, former, or current), pack-years up to SAPALDIA1, pack-years between SAPALDIA1 and -2, cigarettes per day at SAPALDIA1 and -2, atopy, BMI at SAPALDIA1, change in BMI, interaction between the two BMI variables, and baseline PM_10_ exposure.

b Positive estimates indicate attenuation of lung function decline associated with PM_10_ decrease. Negative estimates indicate acceleration of lung function decline with PM_10_ decrease.

c *p*-Value for interaction between change in home outdoor exposure of PM_10_ and genotype parameterized in three different genetic models. The *p*_int_ values presented here represent the most significant (lowest) *p*-value obtained from the three different genetic models. Bonferroni significance level for 12 comparisons [three respiratory function tests (FVC, FEV_1_, FEF_25–75_) × times four association tests], *p* = 0.00417.

d Genotype distribution: *p21* CA, *n* = 594; *p21* AA, *n* = 31.

e Diplotype distribution is labeled as follows: −/−, none of the specific haplotype present; (*rs667515*, *rs9344*)/− (e.g., GG/−), one of the specific haplotypes present; (*rs667515*, *rs9344*)/(*rs667515*, *rs9344*) (e.g., GG/GG), for two of the specific haplotypes present.

**Appendix 1:** The SAPALDIA Team.

Member	Specialty
Study directorate	
U. Ackermann-Liebrich	Epidemiology
J.M. Gaspoz	Cardiology
P. Leuenberger	Pneumology
L.J.S. Liu	Exposure
N.M. Probst Hensch	Epidemiology/genetic and molecular biology
C. Schindler	Statistics
T. Rochat	Pneumology

Scientific team	
J.C. Barthélémy	Cardiology
W. Berger	Genetic and molecular biology
R. Bettschart	Pneumology
A. Bircher	Allergology
G. Bolognini	Pneumology
O. Brändli	Pneumology
M. Brutsche	Pneumology
L. Burdet	Pneumology
M. Frey	Pneumology
M.W. Gerbase	Pneumology
D. Gold	Epidemiology/cardiology/pneumology
W. Karrer	Pneumology
R. Keller	Pneumology
B. Knöpfli	Pneumology
N. Künzli	Epidemiology/exposure
U. Neu	Exposure
L. Nicod	Pneumology
M. Pons	Pneumology
E. Russi	Pneumology
P. Schmid-Grendelmeyer	Allergology
J. Schwartz	Epidemiology
P. Straehl	Exposure
J.M. Tschopp	Pneumology
A. von Eckardstein	Clinical chemistry
J.P. Zellweger	Pneumology
E. Zemp Stutz	Epidemiology

Scientific team at coordinating centers	
P.O. Bridevaux	Pneumology
I. Curjuric	Epidemiology
J. Dratva	Epidemiology
D. Felber Dietrich	Cardiology
D. Keidel	Statistics
M. Imboden	Genetic and molecular biology
H. Phuleria	Exposure
E. Schaffner	Statistics
G.A. Thun	Genetic and molecular biology
